# Cholecystectomy-induced thrombotic microangiopathy (TMA) in a postpartum patient successfully treated with eculizumab: a case report

**DOI:** 10.1186/s13256-024-04804-9

**Published:** 2024-12-26

**Authors:** Ashley Meyer, Kathryn Uchida, Matthew Nguyen, Kenny Vongbunyong, Dong Ren, Ramy Hanna, Minh-Ha Tran, Omar Darwish

**Affiliations:** 1 Department of Internal Medicine, University of California Irvine Medical Center, 333 City Blvd West, Suite 500, Orange, CA 92868 USA; 2 Department of Pathology and Laboratory Medicine, University of California, Irvine, Orange, CA USA

**Keywords:** Atypical hemolytic uremic syndrome, Microangiopathic hemolytic anemia, Thrombotic microangiopathy, Case report

## Abstract

**Background:**

Thrombotic microangiopathy (TMA) is a rare, life-threatening disorder characterized by microangiopathic hemolytic anemia, thrombocytopenia, and end-organ damage. Atypical hemolytic uremic syndrome (aHUS) is even less common, comprising less than 10% of hemolytic uremic syndrome (HUS) cases. aHUS in postpartum is associated with poor maternal outcomes, with the majority of cases resulting in end-stage renal disease. aHUS, unlike other types of TMA, is related to complement dysregulation. Thus, the current treatment of choice for aHUS is complement blockade, which limits unregulated activation of complement and complement-mediated end organ damage.

**Case presentation:**

We present a rare case of a previously healthy, postpartum, 20-year-old Hispanic female patient who underwent a laparoscopic cholecystectomy and subsequently developed complement-mediated TMA, successfully treated with eculizumab. Unique to our case was renal failure owing to multiple insults and partial resolution of hematologic TMA findings prior to initiation of eculizumab.

**Conclusion:**

Our case emphasizes the importance of clinicians possessing a high degree of clinical awareness and judgement surrounding complement-mediated TMA, aHUS and its subsets, and surgery as a precipitator, regardless of safety, particularly during the postpartum period.

## Background

Thrombotic microangiopathy (TMA) is a rare, life-threatening disorder characterized by microangiopathic hemolytic anemia, thrombocytopenia, and end-organ damage. Atypical hemolytic uremic syndrome (aHUS) is even less common, making up less than 10% of hemolytic uremic syndrome (HUS) cases. aHUS in postpartum is especially uncommon, affecting about 1 out of every 25,000 pregnancies. It is associated with poor maternal outcomes, with the majority of cases resulting in end-stage renal disease [1]. TMA can be categorized into two syndromes, thrombotic thrombocytopenic purpura (TTP) and HUS. TTP is classically recognized as the disease that manifest as the pentad (neurological impairment, fever, thrombocytopenia, microangiopathic hemolytic anemia, and acute renal failure) whereas HUS is recognized as a disease marked as the triad (thrombocytopenia, microangiopathic hemolytic anemia, and acute renal failure. Clinically using the pentad versus the triad as criteria to distinguish between the two diseases can be difficult. A more reliable way to differentiate between the two is by checking for ADAMTS13 activity with depressed levels signifying TTP.

HUS can be further divided into typical and atypical HUS. Typical HUS accounts for the majority of HUS cases and occurs after a diarrheal illness, usually *Escherichia coli* O157:H7 or *Shigella dysenteriae*.

aHUS is less common with a broader range of etiology but may present similar to typical HUS. aHUS is often related to complement deficiency, specifically factor H. Factor H is a cofactor in the C3b-inactivating enzyme complement factor I convertase that regulates activation of the alternative C3 complement pathway. Defects in complement regulation can lead to uncontrolled complement activation with resultant endothelial cell damage, which can have thrombotic effects.

Thus, treatment of aHUS mainly focuses on limiting unregulated activation of complement-mediated destruction. Eculizumab is a recombinant humanized monoclonal antibody that inhibits the production of complement protein C5, thus blocking complement-mediated inflammation and cytolysis. Owing to its few documented side effects and no reported drug resistance, eculizumab is recognized as the treatment of choice for aHUS [2, 3].

We report an interesting case of aHUS triggered by cholecystectomy in a previously healthy, postpartum 20-year-old patient with equivocal aHUS genetic testing, and who was successfully treated with eculizumab.

## Case presentation

A previously healthy 20-year-old Hispanic female patient presented 2 months postpartum to an outside hospital with 4 days of abdominal pain and elevations in transaminase and total bilirubin levels. Abdominal ultrasound showed a dilated common bile duct (CBD, 8 mm). Endoscopic retrograde cholangiopancreatography (ERCP) revealed a CBD stone that was removed after large papillotomy. She underwent a laparoscopic cholecystectomy that was complicated by dramatic fall in hemoglobin (Hb) unexplained by a relatively small perihepatic hematoma extending to the gallbladder fossa. On the morning of the cholecystectomy, the Hb and platelet count (PLT) were 12.0 g/dL and 316,000/µL, respectively; on the same day, the immediate post-operative Hb and PLT were 5.4 g/dL and 41,000/µL. Furthermore, acute kidney injury (AKI) developed. Mesenteric angiogram showed no active contrast extravasation and indocyanine green cholangiography confirmed no bile duct injury during the procedure. She was transferred to our hospital for a higher level of care in the medical intensive care unit (MICU) and for further workup of postoperative anemia.

On presentation, she endorsed nausea, fatigue, and abdominal pain of 6/10 severity. She was afebrile with a heart rate of 123 bpm, blood pressure 173/110 mmHg, respiratory rate of 20 breaths per minute, with oxygen saturation 98% on room air. Physical exam revealed a distended and tender abdomen with multiple laparotomy insertion sites covered in gauze, with minimal serosanguineous drainage. She also had a right abdominal drain with serosanguinous blood output. Notable labs on admission include white blood cells (WBC) 19.1 × 10^9^/L, Hb 7.9 mmol/L, mean corpuscular volume (MCV) 84 µm^3^, PLT 86,000/mm^3^, reticulocyte 3.6%, lactate dehydrogenase (LDH) 2042 U/L, haptoglobin < 30 mg/dL, total bilirubin 9.2 mg/dL, direct bilirubin 5.3 mg/dL, blood urea nitrogen (BUN) 63 mg/dL, creatinine 6.5 mg/dL, alkaline phosphatase 60 IU/L, aspartate transaminase (AST) 218 U/L, alanine transaminase (ALT) 439 U/L, and lipase 3480 U/L. Computed tomography (CT) abdomen pelvis showed hyperdense right perihepatic fluid collection measuring up to 1.4 cm in thickness (decreased in size compared with imaging 3 days prior). The patient had minimal urine output secondary to AKI. A quinton was placed and the patient received hemodialysis for volume optimization.

Further workup included a chest x-ray that demonstrated increased bilateral pulmonary opacities suggestive of worsening pulmonary edema as well as bilateral pleural effusions with associated bibasilar atelectasis. A magnetic resonance cholangiopancreatography was performed and showed a stable 10 × 0.7 cm perihepatic hematoma along the anterior and lateral capsular margin of the right hepatic lobe without any evidence of biliary dilation.

**Fig. 1:**
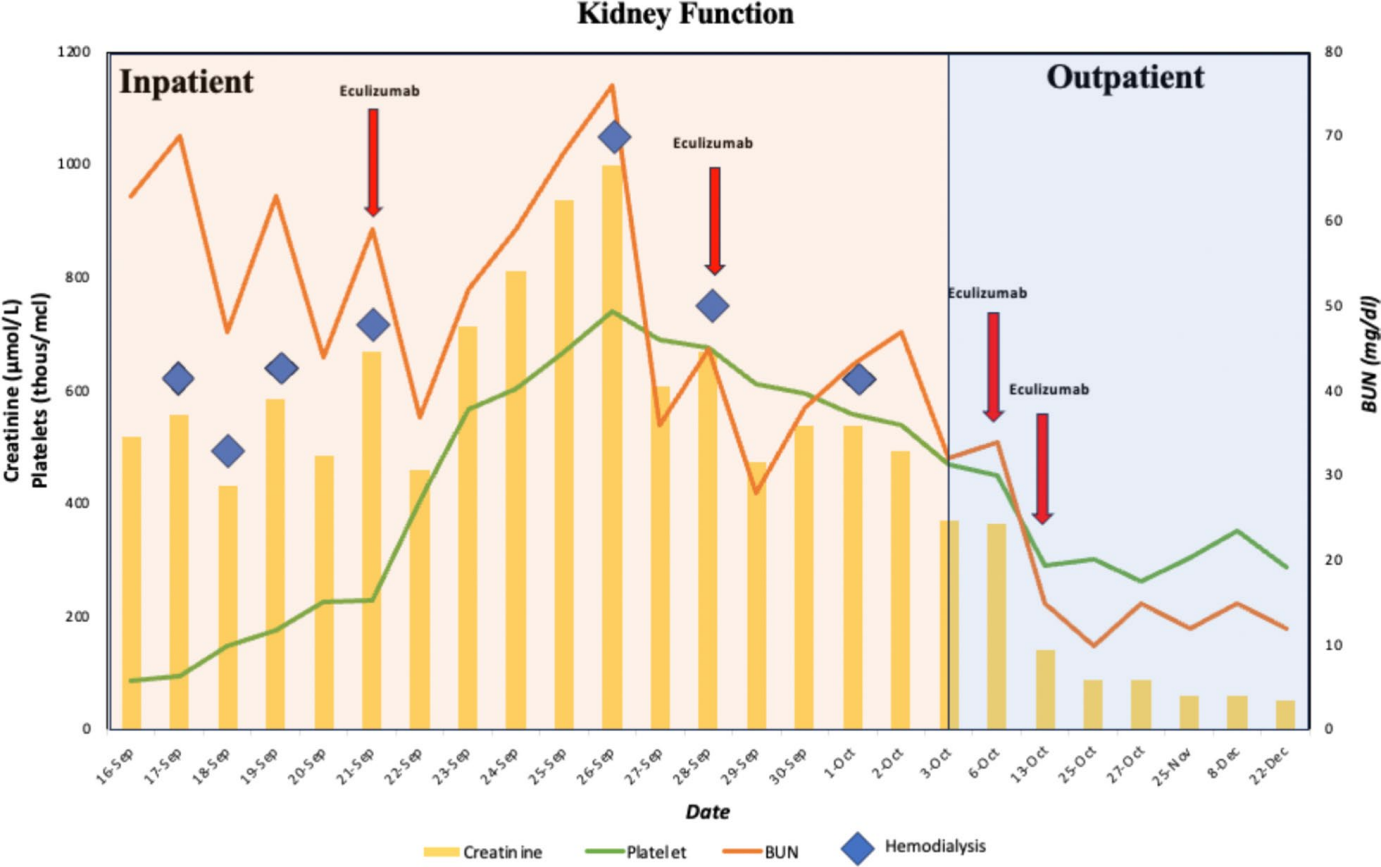
Kidney function as a measure of blood urea nitrogen (BUN), creatinine, platelets

Owing to concern for hemolytic anemia/TMA, a full workup of anemia was done which revealed a negative direct and indirect Coombs test, elevated erythropoietin, and iron panel consistent with anemia of chronic disease. Her vitamin B12, zinc, lead, and copper levels were within normal limits. Testing was negative for human immunodeficiency virus (HIV), hepatitis C virus (HCV), hepatitis B virus (HBV), and coronavirus disease 2019 (COVID-19). Her autoimmune workup also showed non-elevated/normal results for antinuclear antibody (ANA), antineutrophilic cytoplasmic antibody (ANCA), anti-double-stranded deoxyribonucleic acid antibodies (dsDNA), and complement 3 and 4 (c3/c4) levels. Moderate schistocytes were reported on peripheral smear, and she was found to have an ADAMTS13 activity of 44%. Creatinine and kidney function improved with the initiation of hemodialysis as demonstrated in Fig. 1.

During the first 5 days, the patient was found to be hypoxic requiring high flow oxygen and carried an elevated WBC (ranged 15,000–22,100/mcL) with fevers (*T*_max_ 101.8 °F). Her blood and urine cultures were negative. She was given piperacillin/tazobactam to cover for possible pneumonia and/or an intra-abdominal infection given her recent cholecystectomy. However, our overall impression was that she was developing a microangiopathic process.

Given high suspicion for atypical HUS with suspicions of complement mediated TMA (cm-TMA), intravenous eculizumab (10 mg/mL) at 900 mg was started on day 5 of admission and given once weekly. Her fever resolved after a few days and the WBC returned to normal range after 1 week. A genetic mutation panel for aHUS was also obtained and revealed a heterozygous missense variant in exon 25 of the *ADAMTS13 *gene. Additionally, genetic testing revealed that she had a heterozygous, synonymous variant in exon 41 of C3, three polymorphisms in the CFH gene, and was heterozygous for the p.Val62lle polymorphism within the CFH gene. The patient was negative for C5 polymorphisms and CRHR1–CFHR3 homozygous deletion associated with CFH auto-antibodies. Her SC5b-9 complement level was 183 ng/mL (normal range < 250 ng/mL).

**Fig. 2:**
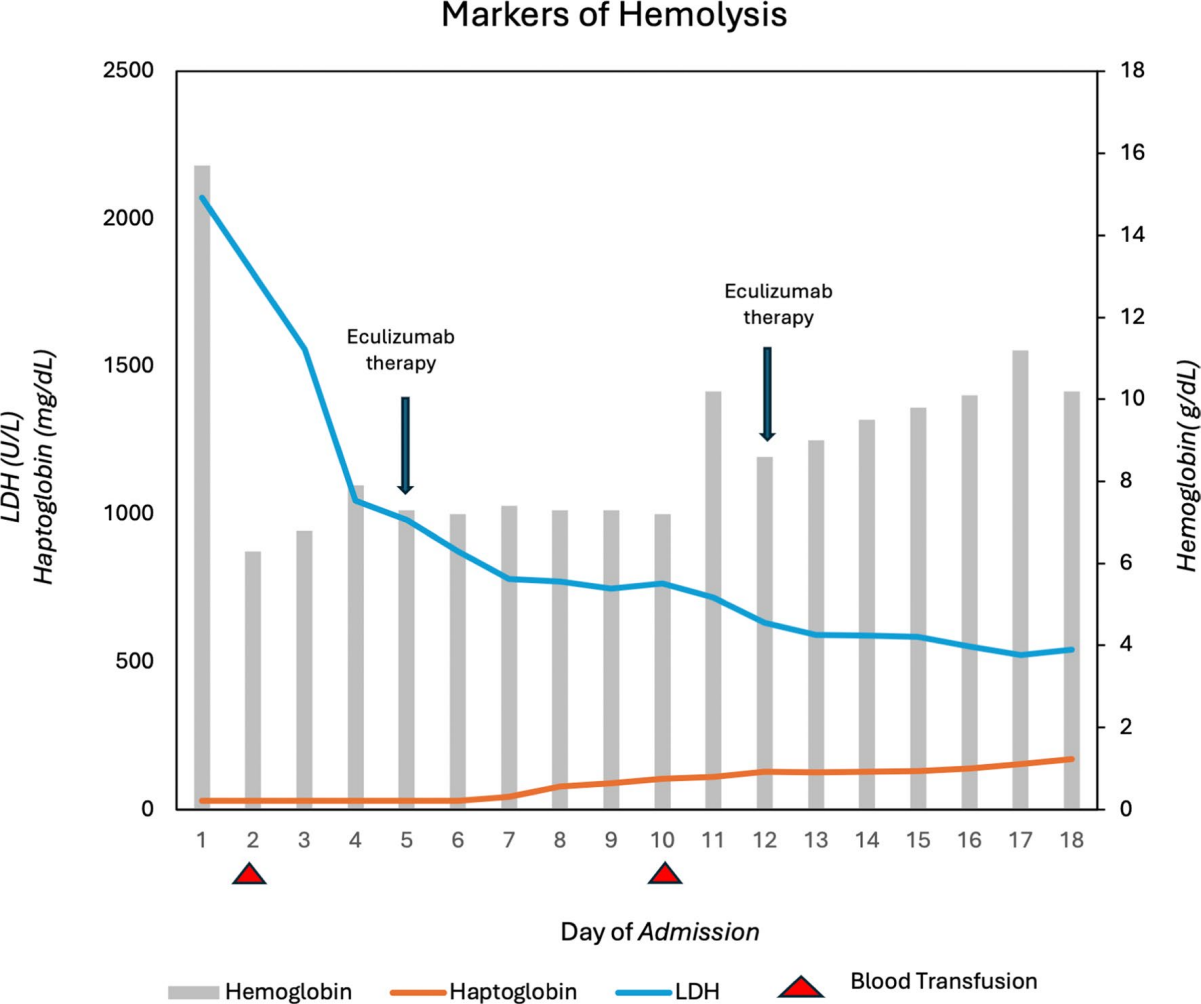
Markers of hemolysis as measured by hemoglobin, haptoglobin, and lactate dehydrogenase

A renal ultrasound was performed on day 5 of admission and showed increased renal parenchymal echogenicity. This was followed by a renal biopsy to help determine if her acute kidney injury could be attributed to ischemia or TMA. Results of her renal biopsy were suggestive of both ATN and TMA as shown in Fig. 2, indicating diffuse acute tubular injury with focal granular casts. The biopsy demonstrated TMA with at least focal active features and evolving chronic changes associated with early focal segmental glomerulosclerosis and focal tubulointerstitial inflammation at the corticomedullary junction with increased eosinophils suggestive of component of allergic tubulointerstitial nephritis. At this time, cm-TMA was confirmed, and a decision was made to continue eculizumab therapy weekly.

The patient experienced no complications while receiving eculizumab therapy. Her clinical condition improved throughout her admission. Prior to discharge, the patient was hemodynamically stable with creatinine level of 3.0 mg/dL, Hb of 11.6 mmol/L, PLT of 336,000/mm^3^, LDH of 331 U/L, alkaline phosphatase of 128 IU/L, ALT of 17 U/L, AST of 31 U/L, and total bilirubin of 1.5 mg/dL. She was discharged on day 20 of admission with a plan to continue hemodialysis twice per week with weekly eculizumab infusions. At outpatient follow-up, the patient had no complaints and demonstrated improvement in renal function. At 2 weeks following discharge, the patient’s permcath was removed and dialysis was discontinued as creatinine remained stable at 1.0 mg/dL. Since then, her creatinine has decreased to 0.7 mg/dl. The patient will continue therapy with eculizumab for 1 year.

## Discussion

Here, we described a rare and unique case of aHUS owing to cm-TMA with cholecystectomy as the apparent driver in a previously healthy, 2-month postpartum patient. Atypical features of this case include a negative cm-TMA genetic panel, pre-C5 blockade normalization of platelet counts and non-elevated sC5b-9 levels. The clinical features suggestive of microangiopathic hemolytic anemia and thrombocytopenia with end-organ damage (oliguric renal failure) prompted rapid initiation of therapeutic complement inhibition. Renal biopsy findings later confirmed clinical suspicion of an underlying TMA and justified continuation of eculizumab therapy as shown in Fig. 2; noticeable improvement ensued. Important aspects of this case include being the first reported case of a complement mediated TMA after a minimally invasive surgery in a healthy, young, 2-month postpartum patient, the presentation of aHUS in the absence of sustained decreased platelet count and the improvement of patient outcomes after being treated with eculizumab.

It has been suggested that the inciting events of cm-TMA can be owing to infections, pregnancies, autoimmune conditions, and high-risk surgeries [4–6]. Laparoscopic cholecystectomy is considered a minimally invasive surgical procedure with approximately 300,000 cholecystectomies performed annually with 1–4% developing complications [7, 8]. Our case is unique as this is the first reported case of a serious complication in an otherwise healthy individual with no previous comorbidities undergoing a minimally invasive procedure that is not generally associated with the development of TMA. This case suggests that minimally invasive surgeries done within the postpartum period could be a precipitator for HUS.

Presence of TTP was excluded given ADAMTS13 levels above 10% (patient’s levels were 44% and 45%) and spontaneous normalization of platelet counts. Disseminated intravascular coagulation (DIC) was also excluded given normal coagulation labs. Our patient’s presentation of aHUS was unique as her platelet levels were mildly decreased if not normal, which is not generally consistent with aHUS. Nonetheless, the remaining findings of renal failure on biopsy with microangiopathic hemolytic anemia were most consistent with aHUS as shown in Fig. 2.

Regarding the pathophysiology of cm-TMA, the relationship between the activation of the complement pathway and thrombotic process involved during microangiopathy has been recently and frequently explored for aHUS. It is proposed that aHUS involves excessive activation of the alternative complement cascade such that the built-in regulators of this system fail to inactivate C3 and C5 convertases and their byproducts, such as C3b and C5b, which then bind to normal healthy cells. C3b also aids in formation of the C5 convertase complex, cleaving C5 to C5a and C5b, which binds to cell membranes thus forming an anchor point for assembly of membrane attack complex (C5b-9). These actions lead to endothelial injury, infiltration of leukocytes and platelets, and result in formation of obstructive thrombi in the vasculature. As a result, these micro thrombi lead to shearing of erythrocytes creating schistocytes and MAHA. Furthermore, the platelet consumption and endothelial injury leads to thrombocytopenia [5]. Van Herpt proposes an interesting case of cm-TMA after undergoing aortic surgery where they hypothesized that the surgical procedure lowered the threshold for unrestrained complement activation and, thus, for complement-mediated TMA to manifest [4]. Compared with the highly invasive aortic surgery, as abovementioned, laparoscopic cholecystectomy is deemed a minimally invasive surgery with minor complications. We believe that our patient’s aHUS presentation was multifactorial driven by her most recent child delivery and possibly surgery lowering the threshold for this patient’s cm-TMA to manifest.

**Fig. 3:**
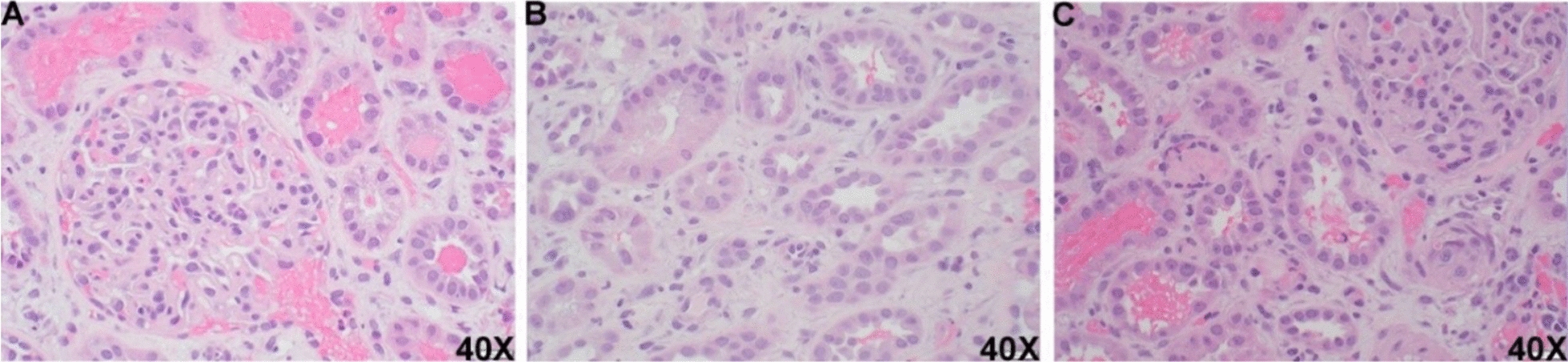
Histologic findings of thrombotic microangiopathy (TMA). **A** Representative section of glomerular ischemic changes, including endothelial swelling and mesangiolysis, and double contours. **B** Representative section of tubular changes, including acute tubular injury and tubular isometric vacuolization. **C** Representative section of arteriolar thrombus and occlusion

Once diagnosis of complement mediated TMA is made, treatment includes targeting the complement proteins to prevent activation of the terminal membrane attack complex with the use of eculizumab. Treatment regarding the length of TMA remains controversial in current literature. It is currently proposed to continue eculizumab treatment at least until resolution of hematologic abnormalities and kidney function, followed by four additional maintenance doses before reevaluating for discontinuation of the medication [9]. At this time, the patient is currently receiving weekly treatment of eculizumab with notable improvement in lab values and prognosis as shown in Fig. 3.

Given the importance of rapidly establishing a steady state for eculizumab and the mortality rates associated with TMA, it is imperative for the clinician to diagnose this condition rapidly. Thus, the presence of multidisciplinary teams (MDT) for TMA has been gaining increased popularity, which has led to a reduction in the response time and total hospitalization [10]. At our institution, the MDT consisted of experts in nephrology, pathology, hematology, radiology, clinical pharmacy, and nursing, which has led to a more rapid response to effectively diagnose the subsets of TMA. In the case of our patient, the rapid diagnosis of cm-TMA led to rapid diagnosis and initiation of treatment, normalization of lab values, and overall improvement of patient outcomes both as inpatient and at outpatient follow-up, further inciting the support for the roles of MDTs for TMA diseases in the inpatient setting.

## Conclusion

Our case emphasizes the importance for clinicians to have a high degree of clinical awareness and judgement surrounding complement-mediated TMA, aHUS and its subsets, and most importantly surgery as a precipitator regardless of relative safety of the procedure, especially during the postpartum period. Although new literature is growing regarding the diagnosis of cm-TMA and the many influencers of TMA, there still exists a paucity of data on the timing and maintenance for the use of C5 blockade. Thereby, this case stresses the importance of exploring the length of treatment for C5 blockades and the establishment of MDTs to address the complexities of TMA.

## Data Availability

Not applicable.

## Abbreviations

TMA: Thrombotic microangiopathy
aHUS: Atypical hemolytic uremic syndrome
HUS: Hemolytic uremic syndrome
TTP: Thrombotic thrombocytopenic purpura
CBD: Common bile duct
mm: Millimeters
g: Grams
dL: Deciliters
µL: Microliters
ERCP: Endoscopic retrograde cholangiopancreatography
MICU: Medical intensive care unit
bpm: Beats per minute
mmHg: Millimeters of mercury
Hb: Hemoglobin
PLT: Platelet count
AKI: Acute kidney injury
WBC: White blood cells
mmol: Millimoles
L: Liters
µm: Micrometers
MCV: Mean corpuscular volume
LDH: Lactate dehydrogenase
BUN: Blood urea nitrogen
mg: Milligrams
AST: Aspartate transaminase
ALT: Alanine transaminase
IU: International units
U: Units
CT: Computed tomography
cm: Centimeters
G6PD: Glucose-6-phosphate dehydrogenase
HIV: Human immunodeficiency virus
HCV: Hepatitis C virus
HBV: Hepatitis B virus
COVID-19: Coronavirus disease
ANA: Antinuclear antibody
ANCA: Antineutrophilic cytoplasmic antibody
dsDNA: Anti-double-stranded deoxyribonucleic acid antibodies
c3: Complement 3
c4: Complement 4
cm-TMA: Complement mediated thrombotic microangiopathy
ng: Nanograms
mL: Milliliters
DIC: Disseminated intravascular coagulation
INR: International normalized ratio
PTT: Partial thromboplastin time
PT: Prothrombin time
MDT: Multidisciplinary teams

## References

[1] Saad AF, Roman J, Wyble A, Pacheco LD. Pregnancy-associated atypical hemolytic-uremic syndrome. AJP Rep. 2016;6(1):e125–8. 10.1055/s-0036-1579539. 26989566 10.1055/s-0036-1579539 PMC4794438

[2] Kaplan BS, Ruebner RL, Spinale JM, Copelovitch L. Current treatment of atypical hemolytic uremic syndrome. Intractable Rare Dis Res. 2014;3(2):34–45. 10.5582/irdr.2014.01001. 25343125 10.5582/irdr.2014.01001 PMC4204535

[3] Raufi AG, Scott S, Darwish O, *et al*. Atypical hemolytic uremic syndrome secondary to lupus nephritis, responsive to eculizumab. Hematol Rep. 2016;8(3):6625. 10.4081/hr.2016.6625. 27781079 10.4081/hr.2016.6625 PMC5062623

[4] van Herpt TTW, Timmermans SAMEG, van Mook WNKA, *et al*. Postsurgical thrombotic microangiopathy and deregulated complement. J Clin Med. 2022;11(9):2501. 10.3390/jcm11092501. 35566627 10.3390/jcm11092501 PMC9100095

[5] Park MH, Caselman N, Ulmer S, Weitz IC. Complement-mediated thrombotic microangiopathy associated with lupus nephritis. Blood Adv. 2018;2(16):2090–4. 10.1182/bloodadvances.2018019596. 30131343 10.1182/bloodadvances.2018019596 PMC6113612

[6] Fakhouri F, Scully M, Provôt F, *et al*. Management of thrombotic microangiopathy in pregnancy and postpartum: report from an international working group. Blood. 2020;136(19):2103–17. 10.1182/blood.2020005221. 32808006 10.1182/blood.2020005221

[7] Blythe J, Herrmann E, Faust D, *et al*. Acute cholecystitis—a cohort study in a real-world clinical setting (REWO study, NCT02796443). Pragmat Obs Res. 2018;9:69–75. 10.2147/POR.S169255. 30498388 10.2147/POR.S169255 PMC6207389

[8] Hassler KR, Collins JT, Philip K, Jones MW. Laparoscopic cholecystectomy. In: *StatPearls*. StatPearls Publishing; 2023. Accessed 17 Nov 2023. http://www.ncbi.nlm.nih.gov/books/NBK448145/. 28846328

[9] Olson SR, Lu E, Sulpizio E, Shatzel JJ, Rueda JF, DeLoughery TG. When to stop eculizumab in complement-mediated thrombotic microangiopathies. Am J Nephrol. 2018;48(2):96–107. 10.1159/000492033. 30110670 10.1159/000492033

[10] Uriol Rivera MG, Cabello Pelegrin S, Ballester Ruiz C, *et al*. Impact of a multidisciplinary team for the management of thrombotic microangiopathy. PLoS ONE. 2018;13(11): e0206558. 10.1371/journal.pone.0206558. 30388144 10.1371/journal.pone.0206558 PMC6214549

